# A dataset on concurrent and immediate retrospective methods for measuring sensory perception and preferences of lemon-flavoured carbonated alcoholic drinks.

**DOI:** 10.1016/j.dib.2022.108346

**Published:** 2022-06-01

**Authors:** Michel Visalli, Takahiro Wakihira, Pascal Schlich

**Affiliations:** a Centre des Sciences du Goût et de l'Alimentation, CNRS, INRAE, Institut Agro, Université de Bourgogne Franche-Comté, Dijon F-21000, France; b INRAE, PROBE Research Infrastructure, ChemoSens Facility, Dijon F-21000, France; c Brewing Science Laboratories, Asahi Quality and Innovations, Ltd., 1-21 Midori 1-Chome, Moriya, Ibaraki 302-0106, Japan

**Keywords:** Sensory analysis, Consumer preferences, Temporal methods, Alcoholic beverage, Check-all-that-apply, Multiple intakes

## Abstract

This article describes a dataset providing temporal sensory descriptions and preferences for four lemon-flavoured carbonated alcoholic drinks. The recruited Japanese consumers (97 men, 96 women) corresponded to the target for this kind of drink: aged between 20 and 40 and regular consumers of flavoured alcoholic drinks. They had to consume a whole can of each drink at home, each on a different day. For sips 1, 4 and 7, they had to check from a check-all-that-apply (CATA) list of eight attributes (alcohol, bitter, carbonated, lemon, refreshing, sour, sweet aroma and sweet taste) that were applicable during three periods of perception – “in mouth before swallowing”, “immediately after swallowing” and “aftertaste”. They were separated into two panels: the consumers in panel SIM (96 consumers) had to do the task simultaneously with the tasting, while the consumers in panel RET (97 consumers) had to do it retrospectively. They also had to rate their liking and report the number of crackers they consumed during the tasting. Once the can had been fully consumed, they had to score their satisfaction level and optionally report comments about the products and the task. The data were used to compare retrospective and concurrent temporal evaluations in a methodologically oriented article entitled “Concurrent vs. immediate retrospective temporal sensory data collection: A case study on lemon-flavoured carbonated alcoholic drinks.” The data could be reused by researchers interested in understanding interactions between alcohol, carbonation, sour, sweet and bitter or to relate temporal perception and preferences for improving product formulation.

## Specifications Table

**Table:** 

Subject	Food science
Specific subject area	Sensory analysis > Temporal perception > Alcoholic drinks
Type of data	TableQuestionnaires
How the data were acquired	Sensory data were acquired by 2 panels of consumers (193 consumers) at home, using the web application TimeSens version 2 [1].
Data format	Tables in raw format (XLSX file)
Description of data collection	Japanese consumers (100 men, 100 women) corresponding to the target of this kind of drink (aged between 20 and 40 and regular consumers of flavoured alcoholic drink) were recruited. They had to consume a whole can of each drink at home, each on a different day. For sip 1, 4 and 7, they had to check from a CATA list of eight attributes (alcohol, bitter, carbonated, lemon, refreshing, sour, sweet aroma and sweet taste) which were applicable during three periods of perception – “in mouth before swallowing”, “immediately after swallowing” and “aftertaste”. They were separated into two panels, and the consumers in panel SIM had to do the task during the tasting, while the consumers in panel RET had to do it retrospectively immediately after the tasting. Ninety-six consumers completed the task in panel SIM and 97 in panel RET. They also had to rate their liking (on a 0–10 visual analogic scale) and report the number of crackers they consumed during the tasting. Once the can had been fully consumed, they had to score their satisfaction level (on a 0–10 visual analogue scale), and optionally report comments about the products and the task (free text).
Data source location	• City/Town/Region: All over Japan • Country: Japan
Data accessibility	Repository name: Mendeley dataData identification number: 10.17632/729tsts8ng.1Direct URL to data: https://data.mendeley.com/datasets/729tsts8ng/1
Related research article	M. Visalli, T. Wakihira, P. Schlich, Concurrent vs. immediate retrospective temporal sensory data collection. A case study on lemon-flavoured carbonated alcoholic drinks. Food Quality and Preference.

## Value of the Data

• These data are useful because they provide information about temporal sensory perception and preferences for lemon-flavoured carbonated alcoholic drinks by consumers over several sips of the full consumption of a can.
• Researchers interested in comparing temporal descriptions of products obtained concurrently and retrospectively to the tasting can benefit from these data.
• Researchers can also use these data to study interactions between flavoured alcoholic drinks key descriptors (alcohol, carbonation, sour, sweet, bitter).
• Product developers can relate temporal perception and preferences to investigate drivers of liking to improve their product formulation.
• Product developers can reproduce the original protocol that allowed us to collect temporal descriptions from consumers using their smartphones, at home, in ecological conditions.
• Sensometricians can reuse data to develop or test new statistical methods for the analysis of discrete time temporal sensory data.

## 1 Data Description

The dataset is provided as an excel file (.xlsx) including 5 sheets.

The sheet “Consumers” provides information about the recruited consumers. “Panel” is the panel to which the consumers have been randomly assigned (RET: retrospective temporal description, SIM: simultaneous temporal description). “Consumer” is the 4-character code of the consumer. The “Panel” and “Consumer” columns are reported in each sheet following this one. “Gender” is the gender of the consumer (male or female). “Age” is the age of the consumer (numeric). “Frequency” is the frequency of drinking of flavoured alcoholic drinks of any type (approximately two or three days a week, approximately four or five days a week or almost every day). “Brand” is the brand that they most frequently drank in the past month (P2, P3, P4, P5 or Other).

The sheet “Drinking mode” contains in column “Answer” the answer to the question Q1: “Please select your drinking mode in the list” (Directly from the can, From a glass without ice, From a glass with ice).

The sheet “Description” contains the CATA sensory descriptions of each consumer for each of the 3 measured sips (1, 4, 7) of the 5 products. “Sip” is the sip position (1, 4 or 7). “Product” is the 2-character code of the lemon-flavoured carbonated alcoholic drink. “Product” is reported in each sheet following this one. “Attribute” is the code of the evaluated descriptor (Carbonated, SweetF for “sweet aroma”, Refreshing, Bitter, Lemon, SweetT for “sweet taste”, Sour, Alcohol). “Period” is the period of the evaluation (T1 for “in mouth before swallowing”, T2 for “immediately after swallowing” and T3 for “aftertaste”). “Score” is 1 if “Attribute” has been checked and thus considered applicable for “Product” by “Consumer” during “Period”, 0 otherwise.

The sheet “Hedonic” contains the liking and satisfaction scores of each consumer for the 5 products. “Sip” is the sip position for liking, (1, 4, or 7). “Attribute” is the code of the evaluated hedonic descriptor (Liking for question Q2: “The sip you just drank: How delicious did you think it was?”, Satisfaction for question Q3: “After having finished the full can, how is your overall satisfaction?”). “Score” takes a numeric value between 0 and 10 (precision=0.01).

The sheet “Crackers” contains in column “Answer” the answer to the question Q5: “How many crackers did you eat?” (numeric value between 0 and 12) for each consumer for the 5 products.

The answers to the free text questions Q4 (“From the first sip to the last one, have you perceived any change in your sensations”) and Q6 (“Please tell us what you felt about this tasting survey”) have not been included in the dataset because of the number of missing answers.

Table 1 displays information about the products. Alcohol content and lemon juice content were obtained from the product package. Brix, acidity, pH, limonene and citral were analysed in the Asahi Group R&D Center. ABV=alcohol by volume, ppm=parts per million.

**Table 1:** Information about products.

Sample	Alcohol content (% ABV)	Lemon juice content (%)	Brix (%)	Acidity (g/100 ml)	pH	Limonene (ppm)	Citral (ppm)
P2	6	3	6.07	0.41	3.43	2.3	0.05
P3	4	14	4.82	0.53	3.44	43.6	1.36
P4	5	10	6.33	0.52	2.88	28.4	0.08
P5	5	1.6	2.63	0.49	3.65	3.7	0.13

Questionnaires 1 and 2 include screenshots of the online questionnaire (TimeSens V2 web app) displayed to RET (questionnaire 2) and SIM (questionnaire 2). The questionnaires have been translated from Japanese.

## 2 Experimental Design, Materials and Methods

### 2.1 Samples

The five products (P1, P2, P3, P4, and P5) were 350 ml cans of commercial lemon-flavoured carbonated drinks. P1 was a nonalcoholic drink that served as a warm-up. P2 to P5 were white liquor-based (Japanese Shochu or Vodka) alcoholic drinks, referred to as “Chu-hai” in Japanese.

The cans were purchased at stores and sent from a research agency to the consumers’ houses. The participants were instructed to put the products in the refrigerator for five or more hours after they had received them and, to the greatest extent possible, to taste the products at the same temperature. To accompany drinks, they also received plain, unsalted crackers. The cans were blinded by white-colored masking films and coded using three-digit labels, and they were presented according to a Williams Latin square, but P1 was served first to every consumer.

### 2.2 Consumers

Two hundred consumers aged 20–39, who were regular consumers of lemon-flavoured carbonated alcoholic beverages, were recruited through an online questionnaire from a panel of consumers belonging to a research agency in Japan. “Regular” referred to consumers drinking flavoured carbonated alcoholic beverages with a frequency of at least twice a week and lemon-flavoured carbonated alcoholic beverages at least once a month. The design of the test was explained to consumers in the online questionnaire. They were informed they would do the test on their smartphones, and they had to sign a consent form to participate in the study. They were financially compensated for their participation. The consumers were separated into two panels that were balanced in their composition (gender, age, frequency of consumption). Panel RET had to evaluate the products retrospectively to the tasting just after they declared that they no longer perceived anything, while panel SIM evaluated them concurrently to the tasting. One hundred and ninety-three consumers finally participated, 97 consumers in panel RET, and 96 in panel SIM.

### 2.3 Descriptors

The descriptors were chosen according to the expertise of Asahi. The same list of descriptors was provided for both the RET and SIM panels: Alcohol, Bitter, Carbonated, Lemon, Refreshing, Sour, Sweet aroma and Sweet taste. (In Japan, sweet aroma is used when it is not possible to describe detailed quality of sweetness in terms of aroma. Sweet aroma can include different types of aromas, such as fruity, floral, caramel, vanilla, honey, etc.) The descriptors were presented as a check-all-that-apply (CATA) list in a random order on the screen, but this order was constant for each consumer across evaluations. No definitions of the descriptors were given to the consumers.

### 2.4 First Connection and Description of the Overall Procedure

Consumers received an email containing an individualized URL to invite them to connect to the web application using their smartphones. The consumers were reminded they had to fully consume each can, to drink one a day, and to use their smartphones for the test. Then, they were asked about their drinking mode (Q1) and invited to keep their drinking mode constant for all the products.

### 2.5 Procedure for Tasting, Repeated on Each Day

Before each tasting of a new product (on each day), the general instructions were reminded. Then, a training exercise presenting the list of attributes (CATA list) was proposed. Then, the procedure for the full consumption of the can and for the evaluation of each sip was reminded.

### 2.6 Tasting: Sips 1, 4 and 7

First, the consumers were invited to take a sip without swallowing.

For panel RET, during eight seconds (T1), the consumers were instructed not to swallow while focusing on perceived sensations and memorizing them. Then, during two seconds, they were invited to swallow. Then, during eighteen seconds (T2), they were instructed to focus on perceived sensations and memorize them. Then, until they do no longer perceived anything (T3), they were instructed to focus on perceived sensations and memorize them. Then, with no time limit, they had to select in three CATA lists all the sensations that applied during each period (T1, T2, T3).

For panel SIM, during eight seconds (T1), the consumers had to select in a CATA list all the sensations that applied. Then, during two seconds, they were invited to swallow. Then, during eight seconds (T2), they had to select in a CATA list all the sensations that applied. Then, during ten seconds, they were invited to wait. Then, until they declared no longer perceiving anything (T3), they had to select in a CATA list all the sensations that applied.

Finally, the consumers had to rate their liking (Q2) on a 0–10 continuous linear scale.

### 2.7 Tasting: Sips 2, 3, 5 and 6

On sips 2, 3, 5 and 6, there was no evaluation (neither CATA nor liking), but the consumers were invited to eat crackers if they wanted.

### 2.8 Tasting: Drinking of the Rest of the Can

After the 7th sip, the consumers were invited to finish the can. Then, they had to rate their overall satisfaction (Q3) using a 0–10 continuous linear scale. Then, a free answer question was asked about their perception of changes in their sensations over time (Q4). Then, the consumers were requested to declare the number of crackers they consumed (Q5). This ended the session for the four first products. After the fifth and last product, the consumers were also asked about their feeling for the difficulty of the questionnaire (Q6).

## Ethics Statements

Each participant was informed of the conditions for participating and validated a consent form.

## CRediT authorship contribution statement

**Michel Visalli:** Conceptualization, Methodology, Software, Validation, Formal analysis, Data curation, Writing – original draft, Visualization. **Takahiro Wakihira:** Conceptualization, Methodology, Validation, Data curation, Resources, Project administration, Investigation, Writing – review & editing. **Pascal Schlich:** Conceptualization, Methodology, Software, Supervision, Funding acquisition, Writing – review & editing.

## Declaration of Competing Interest

The authors declare that they have no known competing financial interests or personal relationships that could have appeared to influence the work reported in this paper.

## Data Availability

A dataset on concurrent and immediate retrospective methods for measuring sensory perception and preferences of lemon-flavoured carbonated alcoholic drinks (Original data) (Mendeley Data).
